# A new acoustic coupling fluid with ability to reduce ultrasound imaging artefacts in brain tumour surgery—a phase I study

**DOI:** 10.1007/s00701-019-03945-x

**Published:** 2019-05-18

**Authors:** Geirmund Unsgård, Lisa Millgård Sagberg, Sébastien Müller, Tormod Selbekk

**Affiliations:** 1 Department of Neuromedicine and Movement Science, Norwegian University of Science and Technology, Trondheim, Norway; 2 Department of Neurosurgery, St. Olav University Hospital, Trondheim, Norway; 3 Norwegian National Advisory Unit for Ultrasound and Image guided therapy, Trondheim, Norway; 4 Department of Public Health and Nursing, Norwegian University of Science and Technology, Trondheim, Norway; 5 Department of Health Research, SINTEF, Trondheim, Norway

**Keywords:** Ultrasound artefacts, Ultrasound image quality, Acoustic coupling fluid (ACF), Artefact reduction

## Abstract

**Background:**

A novel acoustic coupling fluid (ACF), with the potential to reduce surgically induced image artefacts during intraoperative ultrasound imaging in brain tumour surgery, has been evaluated with respect to image quality and safety in a clinical phase 1 study.

**Methods:**

Fifteen patients with glioblastoma (WHO grade IV) were included. All adverse events were registered in a 6-month study period. During acquisition of 3D ultrasound image volumes, three different concentrations of the ACF and Ringer’s solution were filled into the resection cavity. The effect of ACF on the ultrasound images was rated by the operating surgeon, and by five independent neurosurgeons evaluating a pair of blinded images from all patients. Images from all patients were analysed by comparing pixel brightness in a noise-affected region and a reference region.

**Results:**

The operating surgeon deemed the ACF images to have less noise than images obtained with Ringers’s solution. The blinded evaluations by the independent neurosurgeons were significantly in favour of ACF (*p* < 0.0001). The analyses of pixel intensities showed that the ACF images had lower amount of noise than images obtained with Ringer’s solution. No radiological sign of inflammation nor circulatory changes was found in the early postoperative MR images. Of the nine complications registered as serious events in the study period, none was deemed to be caused by the ACF.

**Conclusion:**

The ultrasound (US) images obtained using ACF have significantly less noise than US images obtained with Ringer’s solution. The rate of adverse events was comparable to what has been reported for similar groups of patients.

## Introduction

Prognosis in patients with primary brain tumours depends on histological classification where glioblastoma patients have a median survival reported from 9 to 17 months [14, 16]. Tools for enhancing surgical resection of brain tumours, in particular gliomas, are increasing [12, 13, 17, 19]. There seems to be an agreement that achieving extensive resections, when done safely without jeopardising neurological function, is important since it improves survival both for high-grade and low-grade gliomas [6, 12, 15].

Ultrasound is currently used as a tool for providing intraoperative 2D or 3D images for the purpose of tumour localization and resection control. For the use in resection control, the resection cavity is filled with saline or Ringer’s solution to provide acoustic coupling between the ultrasound transducer and tissue. However, attenuation of acoustic waves is very low in water-based irrigation fluids (saline, Ringer’s solution) compared to the brain. This difference in attenuation is the cause of artefacts that may severely degrade the ultrasound images. These artefacts are seen as high-intensity signals at the resection cavity wall and beyond, potentially masking a small tumour remnant and generally make the image interpretation more difficult [11].

The cross-disciplinary research group in the Norwegian National Advisory Unit for Ultrasound and Image-guided Therapy at St. Olavs Hospital Trondheim, Norway, has developed a fluid (ACF) intended for use in the resection cavity during ultrasound imaging. The purpose of the acoustic fluid is to reduce noise in the ultrasound images, and thereby provide images with better ultrasound image quality, which may provide a better basis for clinical decision making and make it easier to detect small tumour remnants near the end of an operation.

The ACF’s effect on image quality was first tested in laboratory measurements using phantoms or fresh animal cadavers. Safety has previously been assessed through two animal studies [2]. In the first pre-clinical study, we injected the acoustic fluid subcortically in eight rats. The rat brains were harvested 9 days later, and there were normal histopathological findings including immunohistochemistry. In a second preclinical study with six pigs, we injected the fluid in the cerebrospinal fluid circulation. After 13–16 days, tissue sampling of dura and brain parenchyma both above and below the tentorium was performed. Microscopic analysis and immunohistochemistry did not reveal any sign of inflammation or acute cellular response, neither in meninges, nor in brain tissue or vessels. Furthermore, no gliosis nor microglial response was observed. There were no signs of seizure or seizure activity on EEG, and in addition, there were no clinical suspicion of altered neurological function.

A clinical phase I study was thereafter designed, approved and performed to assess safety and efficacy of the new acoustic coupling fluid in surgery of 15 patients with glioblastoma. This paper summarises the results obtained regarding the effect of the fluid on image quality, and the preliminary assessment of patient safety. The main objective of the image analysis was to compare the image quality obtained using three different concentrations of the novel acoustic coupling fluid with the image quality obtained using Ringer’s solution. The patient safety was assessed by clinical variables recorded prior to and during the intervention, as well as in postoperative controls within 24–72 h, 1 month and 6 months.

## Methods and material

### Study design and objectives

The clinical study is a single-centre prospective study, performed at the department of Neurosurgery, St. Olav University Hospital, Trondheim, Norway. The study was approved by the regional ethical committee (REC Central, approval 2012/1266) and Norwegian Medicines Agency (EudraCT No. 2012-005567-27). Informed consent was obtained from all individual participants included in the study.

Sponsor of the study was the Norwegian National Advisory Unit for Ultrasound and Image-guided Therapy (USIGT) at St. Olav University Hospital.

The objective of the clinical study was to test the fluid during surgery for histopathologically proven glioblastoma to assess safety and efficacy on image quality. The study was not designed to test the effect on the extent of the resection.

### Study population and baseline characteristics

The study aimed to include 15 patients, male and female > 18 years undergoing resection for glioblastoma. The exclusion criteria included hypersensitivity to egg protein, peanut protein, soy protein, glycerol and allergies to diary and marine products.

The first patient was included in October 2013, while the last examination of the last patient included in the study was performed on May 3, 2016. A total of 18 patients gave their consent to be included in the clinical study. In three patients, the biopsies taken early in the surgery did not confirm a histopathology of glioblastoma. These three patients were therefore not included in accordance with the study protocol, and the ACF was not used during surgery.

In Table 1 the patient demographics and preoperative status is shown. The patients enrolled in the study had a male domination and a high percentage of tumours located in the left brain hemisphere (73%).

**Table 1:** Patient demographics and preoperative clinical status

Age in years (mean ± SD)	64 ± 7
Gender, *n* (%)	
Male	11 (73)
Female	4 (27)
Preoperative symptoms^*^, *n* (%)	
Headache	4 (27)
Seizures	5 (33)
Cognitive symptoms	5 (33)
Unsteadiness/ataxia	5 (33)
Visual disturbances	1 (7)
Aphasia/dysphasia	7 (47)
Cranial nerve deficits	2 (13)
Motor deficits	2 (13)
NIH Stroke Scale ≥ 2, *n* (%)	2 (13)
Mini Mental Status ≤ 28, *n* (%)	7 (47)
Karnofsky Performance Status ≥ 70, *n* (%)	14 (93)
American Society of Anaesthesiology score (ASA score), *n* (%)	
1–2	12 (80)
≥ 3	3 (20)
Tumour location*, *n* (%)	
Multifocal	2 (13)
Frontal	9 (60)
Temporal	5 (33)
Parietal	6 (40)
Occipital	0 (0)
Insula/basal ganglia	2 (13)
MRI findings	
Contrast enhancement/ring on MRI, *n* (%)	15 (100)
Tumour crossing midline, *n* (%)	2 (13)
Growth into ventricular system, *n* (%)	5 (33)
Eloquent location (Sawaya grade 3), *n* (%)	9 (60)
Left-sided tumour, *n* (%)	11 (73)
Preoperative tumour volume, cm^3^, mean ± SD	35 ± 25
Max depth from craniotomy, mm, median ± IQR**	55 ± 17
Largest diameter oedema, mm, median ± IQR**	70 ± 26
Midline shift, mm, median ± IQR**	3 ± 4
Primary operation, *n* (%)	14 (93)

^*^Multiple symptoms/locations can be present for a patient

^**^Interquartile range

### Fluids and equipment

The ACF is a sterile mixture consisting of glycerol, vegetable oil, emulsifier and sodium chloride. The testing of the novel ACF involved 3 different dilution of the acoustic attenuating components; ACF-1, ACF-2 and ACF-3. The three different concentrations of ACF was produced by the hospital pharmacy (Sykehusapoteket, St. Olavs Hospital, Trondheim) on the day of surgery.

The ultrasound data were obtained at different stages (two or three) of the surgery, using Ringer’s solution and ACF. The ultrasound acquisition was obtained with the SonoWand Invite system (Sonowand AS, Trondheim, Norway), using a flat linear probe (12 FLA) with frequency range 6–12 MHz. The SonoWand Invite system combines ultrasound imaging with neuronavigation technology, enabling acquisition of 3D ultrasound image volumes during surgery [19]. Navigated intraoperative ultrasound data may be presented as overlays on the preoperative MR data, side-by-side or as 3D US volumes alone. The surgeon is able to navigate seamlessly in 3D US volume. The rapid and flexible image acquisition makes it possible to make repeated ultrasound acquisitions during an operation.

### Administration of the fluids

After partial removal of the tumour, the fluids were filled into the resection cavity just prior to ultrasound image acquisition using a 50 ml syringe. After the 3D US acquisitions, the respective fluid was removed using a suction device. The ACF has a white colour. After each ultrasound acquisition with ACF, the fluid was sucked out and the cavity was flushed with Ringer’s solution until there was no discolouration of the Ringer. A laboratory dilution test showed that even by a dilution with water to 0.25% ACF, a considerable discolouration was still observed. Flushing until no discolouration is seen should ensure close to complete removal of ACF after US imaging.

### Safety - clinical variables

Safety was assessed by registration of adverse events and by dedicated clinical tests and postoperative MR imaging. Any serious adverse events that occurred throughout the study was reported to the legal authorities (Statens Legemiddelverk) according to ICH/GCP-guidelines.

Research nurses involved in the study were collecting clinical data from the patients, both through clinical examinations and from electronic medical records by using validated outcome measures. In addition, included patients were scheduled for controls as outpatients to both the principal investigator/surgeon (GU) and a research nurse at 1 and 6 months. MRI-images were controlled and assessed by an experienced neuro radiologist. The core patient safety parameters investigated in the study were inflammations of the brain tissue, development of hydrocephalus and epileptic seizures. All detectable adverse events, both during operation as well as events that were registered in the medical journal in the postoperative period, were registered by the research nurses. Serious adverse events (SAEs) and suspected unexpected serious adverse reaction (SUSARs) were reported to the legal authorities (Statens Legemiddelverk).

Clinical variables acquired pre- and postoperatively (1–3 days prior to surgery; 24–72 h, 30 days and 6 months postoperatively) included Karnofsky performance status, Charlson comorbidity index, Glasgow Coma Scale, Mini Mental Status and NIH Stroke scale. Patient-reported quality of life (EQ-5D 3L) was acquired by self-registration before surgery and telephone controls (at 1 and 6 months). MR T1-weighted images were obtained prior to surgery (1–3 days), early postoperative (24–72 h after surgery), at 1 month and at 6 months. Diffusion-weighted images were acquired early postoperatively in all patients. The postoperative MR images were evaluated with respect to any indications of inflammation in the brain or meningitis, significant circulatory changes or hydrocephalus.

### Image evaluation and analyses

The qualitative assessment of the ultrasound images obtained by using the different fluids in the resection cavity was done both in the operating room by the operating surgeon and postoperatively by five independent neurosurgeons.

In the operating room, the ultrasound images acquired using the different fluids in the resection cavity were displayed on the SonoWand system for visual inspection. Each of the image volumes was individually rated with respect to the degree of image artefacts according to a three-level grading (none – some – much). In addition, the surgeon rated which of the image volumes acquired with the different fluids that provided the overall best image quality with respect to delineation of anatomy and level of noise.

The postoperative assessment of the ultrasound images was performed in a blinded fashion by five consultant neurosurgeons. One image obtained with Ringer’s solution and one image obtained with ACF in the resection cavity was presented in arbitrary order from each patient and rated using a 10-point scale (1 = least, 10 = best). The two images were obtained consecutively with only a few minutes interval. The five observers rated the images by answering the three questions:

1. How easy is it to see the difference between tumour tissue and its surrounding brain tissue in the ultrasound image?
  (provided that the tumour is not completely resected)
2. How easy is it to interpret the image in the region below the resection cavity in the ultrasound image?
  (provided a resection cavity is present)
3. How easy is it to use the image to identify remaining tumour tissue (for resection)?

The quantitative image analyses were performed postoperatively by a single operator blinded for the results of the qualitative image analyses using the software 3DSlicer 4.4.0 and Matlab 9.0.0 (MathWorks).

**Fig. 1:**
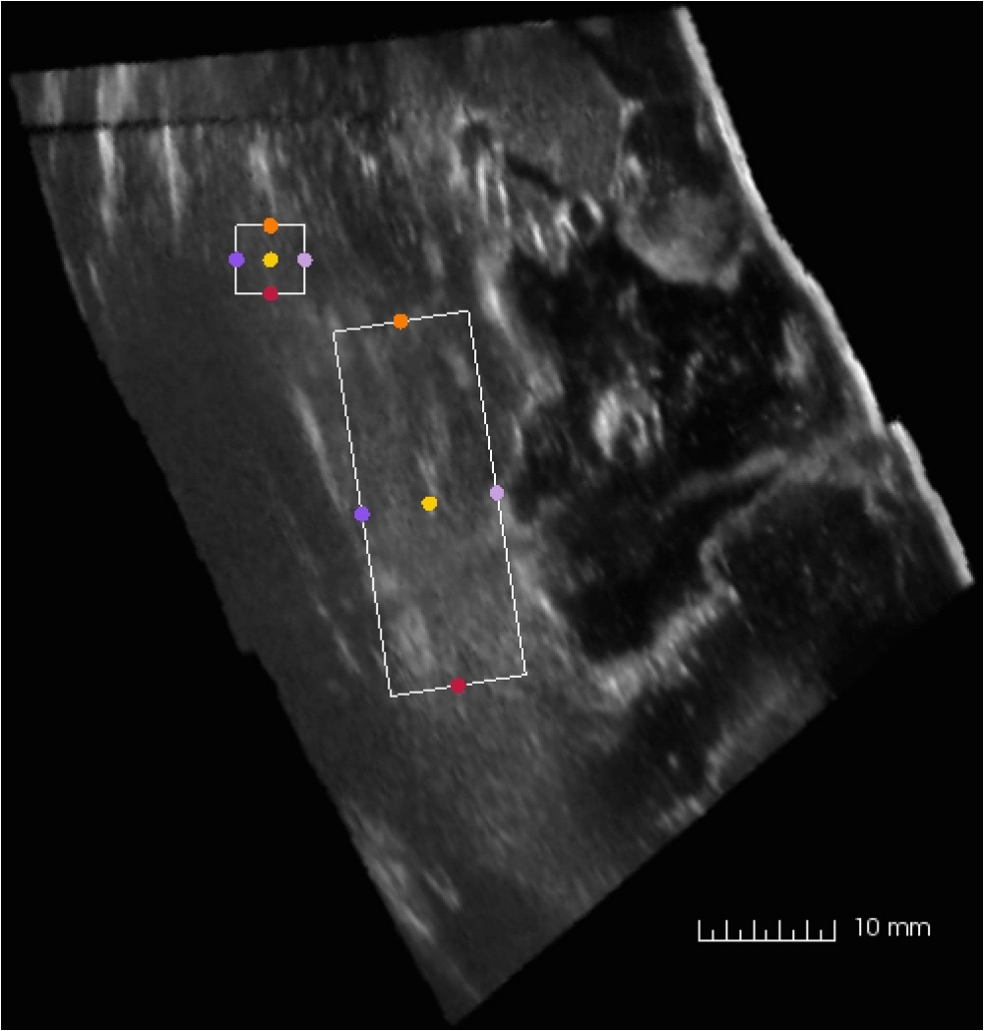
Image slice extracted from a 3D US volume, with the two regions of interest (ROIs) selected for analysis of median intensities. The smaller square indicates the reference region (#2), in which similar tissue as below the resection cavity is expected. The larger rectangle (#1) is selected to be directly below the resection cavity, in an area where enhancement artefacts are expected to be present

Two different regions of interests (ROI) were selected for analyses in each of the acquired image volumes. One region (30 × 10 × 10 mm) was selected below the resection cavity in the image volume, while the other region (5 × 5 × 5 mm) was selected at similar depth but lateral for the resection cavity and served as a reference region for the expected intensities in the ultrasound image volume as shown in Fig. 1. The operator strived to obtain a reference region in tissue that were expected to be similar as the tissue below the resection cavity, as deemed from the preoperative MR T1 images. That is, if the tissue below the resection cavity was oedematous, the reference region was preferably selected in oedematous tissue and at the same depth, if possible. The median brightness of the pixels in the respectively ROIs was calculated. The fluid providing a median intensity of the ROI #1 being closest to the median intensity of the reference ROI #2, was interpreted to be the fluid providing the most correct representation of tissue in the ultrasound images.

### Statistical methods

SPSS Statistics was used for descriptive data analyses. QQ-plots were used to test for normal distribution. Mean ± SD are presented if data is normally distributed while medians and interquartile range (IQR) are presented if data are skewed. Kaplan-Meier was used for survival curve. Independent samples t-test was used for the quantitative image evaluations, comparing the difference in intensity of the two selected ROIs in the images acquired with different fluids in the resection cavity. The Pearson’s chi-squared test was used for evaluation of differences in rating of the images by the surgeon in the operating room.

The postoperative qualitative image evaluation involving five raters and three questions was analysed by using a repeated measures ANOVA (analysis of variance) statistical model.

## Results

Ultrasound images with Ringer or ACF in the resection cavity could be successfully obtained from all the included 15 glioblastoma patients and both qualitative and quantitative assessment of the images were performed.

### Qualitative assessment of the fluids’ impact on image quality

**Fig. 2:**
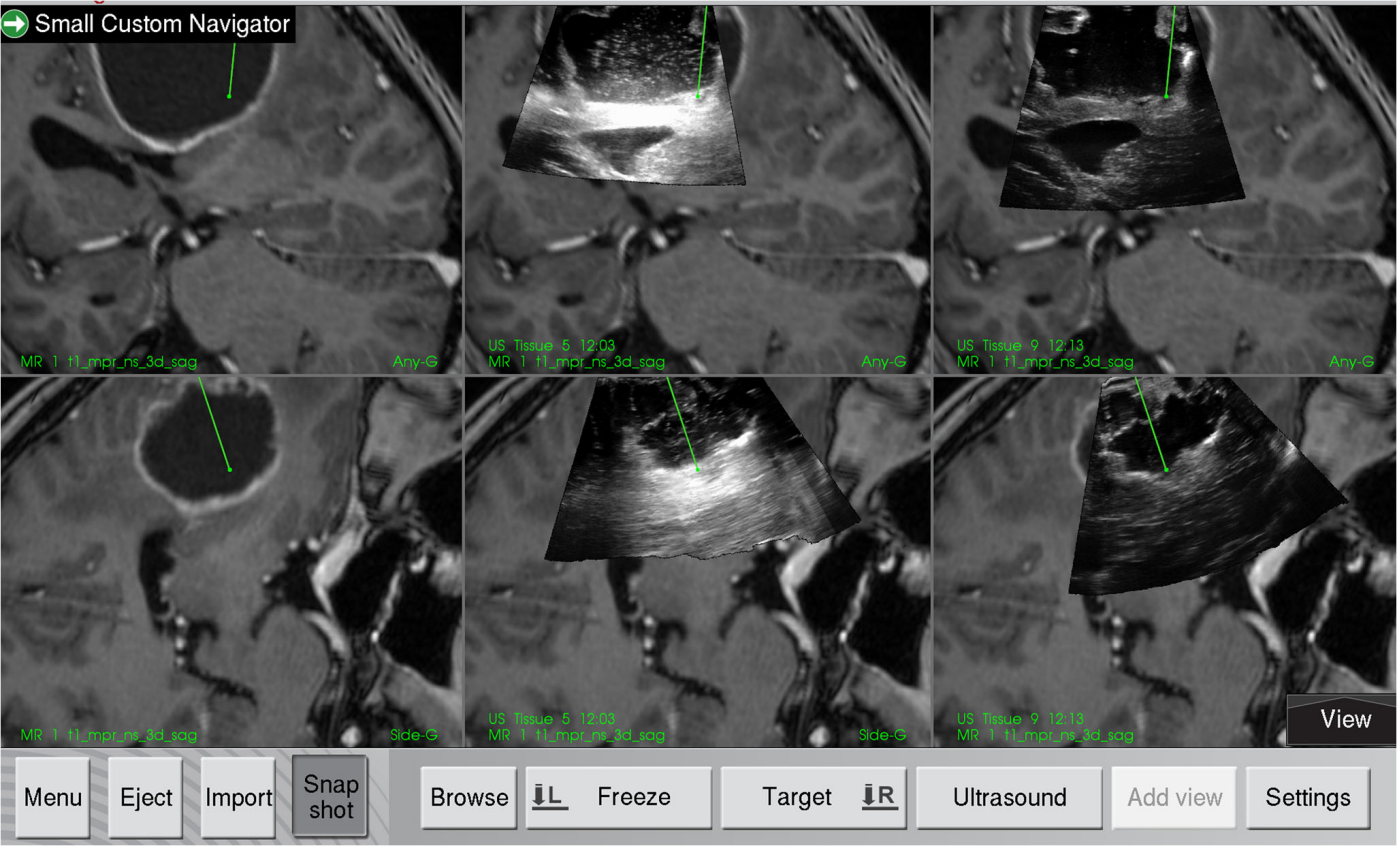
Display from the navigation system, showing two perpendicular image slices extracted from the MRI or 3D ultrasound volume according to the orientation of the navigated tool. The preoperative MR T1-weighted contrast image is shown to the left. The middle column shows the ultrasound images acquired with Ringer’s solution in the resection cavity, and in the right column, the ultrasound obtained with ACF-3 is shown overlaid the MR images

The ultrasound data acquired with different fluids was displayed on the Sonowand Invite system for qualitative interpretation by the operating surgeon. The 3D image volumes were typically displayed as two image slices for each volume as shown in Fig. 2. A navigation pointer was used to inspect the image volumes on the navigation display, with the image slices being extracted on basis of the position and orientation of the navigation pointer.

### Intraoperative assessment by the operating neurosurgeon

The operating neurosurgeon (GU) assessed the ultrasound images obtained with Ringer’s solution in the resection cavity to contain more image artefacts than images obtained with any of the variants of ACF. The chi-square test showed a significantly higher noise level in the Ringer images compared to the ACF images (*p* < 0.05).

The operating surgeons were also asked to choose the image with the best image quality.

The ACF-2 had the highest success rate (# wins), as it was rated to have the best image quality in 19 of 29 test sequences (65.5%). There was no significant difference in the # wins between the three concentrations of ACF. Ringer’s solution had equal quality with the ACF in 1 of 31 test rounds (3.2%).

### Postoperative blinded rating by independent neurosurgeons

A pair of images with Ringer’s solution and ACF in random order from each of the 15 operations was evaluated by five independent neurosurgeons. The neurosurgeons were asked to answer three questions for each pair. Table 2 lists the evaluations for all three questions across the 15 image pairs and the five raters. The results were significantly in favour of ACF (*p* < 0.0001). For question 3, “How easy is it to use the image to identify remaining tumour tissue (for resection)?” the mean difference ACF–Ringer’s was 3.03 (2.39, 3.66). The difference between ACF and Ringer’s solution was significant (*p* < 0.00001) for all five raters.

**Table 2:** Score of ACF vs. Ringer’s solution for the 15 images (across 5 raters and 3 questions)

Included Patient No.	Mean score (SD) Ringer	Mean score (SD) ACF	Difference ACF - Ringer (95% CI)	*p* value
1	2.80 (1.37)	8.00 (0.53)	5.20 (4.42, 5.98)	< 0.0001
2	3.73 (1.71)	7.73 (0.70)	4.00 (3.02, 4.83)	< 0.0001
3	3.40 (2.10)	3.40 (2.29)	0.00 (− 1.64, 1.64)	1.00
4	2.60 (1.64)	7.07 (0.96)	4.47 (3.46, 5.47)	< 0.0001
5	4.87 (1.81)	7.33 (0.72)	2.47 (1.43, 3.50)	< 0.0001
6	4.60 (1.59)	7.20 (1.74)	2.60 (1.35, 3.85)	0.0002
7	5.33 (2.32)	6.93 (1.28)	1.60 (0.20, 3.00)	0.0267
8	3.73 (1.94)	6.53 (1.92)	2.80 (1.35, 4.25)	0.0005
9	4.67 (1.88)	6.87 (1.13)	2.20 (1.04, 3.36)	0.0006
10	6.27 (1.71)	7.20 (1.26)	0.93 (− 0.19, 2.06)	0.10
11	5.20 (2.08)	7.22 (0.82)	2.13 (0.96, 3.31)	0.0009
12	4.00 (2.07)	7.87 (0.92)	3.87 (2.67, 5.06)	< 0.0001
13	4.80 (1.97)	7.80 (0.68)	3.00 (1.90, 4.10)	< 0.0001
14	3.20 (2.40)	7.20 (1.15)	4.00 (2.59, 5.41)	< 0.0001
15	4.00 (2.04)	9.00 (0.85)	5.00 (3.83, 6.17)	< 0.0001

As seen in Table 2, there is some heterogeneity in the judgement of image quality (ACF–Ringer) across the 15 images, with no improvement seen in the images from patient 3, and a non-statistically significant difference (*p* = 0.10) of less than 1 unit seen in the images from patient 10. For the other 13 patients, the differences between ACF and Ringer are all statistically significant, with values ranging between 1.60 and 5.20.

**Fig. 3:**
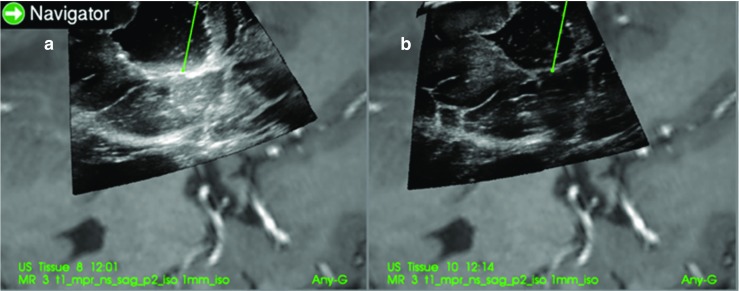
(patient 2). Image **a** is with Ringer’s solution in the cavity and image **b** is with ACF. At the tip of the navigation pointer (green dot), there are artefacts in **a** with the same intensity as remaining tumour tissue, which is absent in **b**

**Fig. 4:**
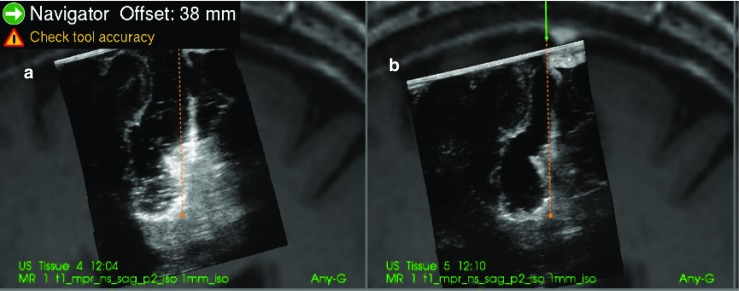
(patient 6). Image **a** is with Ringer’s solution and **b** is with ACF in the operation cavity. The artefact signals in **a** makes it difficult to do a safe judgement of tumour borders. In **b**, the yellow marker in the image is pointing at signals that are interpreted as tumour at the bottom of the cavity

**Fig. 5:**
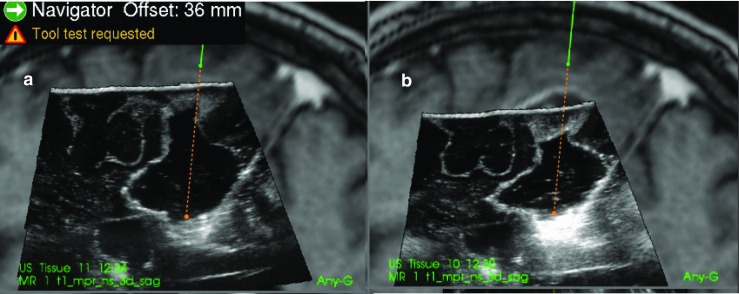
(patient 13). **a** With ACF and **b** with Ringer’s solution in the operation cavity. In **b**, it is difficult to interpret the image in the region below the operation cavity because of artefacts

Examples of the ultrasound images that were evaluated for patient 2, 6 and 13 are shown in Figs. 3, 4 and 5.

**Fig. 6:**
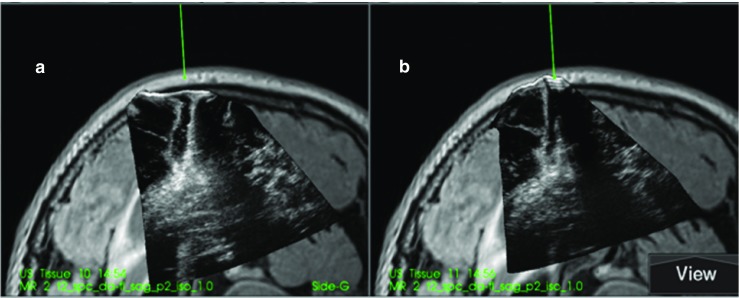
(patient 3). Image **a** is with Ringer’s solution and **b** is with ACF in the operation cavity. Since the cavity is narrow and the sound waves mostly pass through brain tissue, there is no enhancement artefacts, and the tumour below the cavity is shown in both images

For the two patients where the neurosurgeons found no difference between ACF and Ringer’s solution the cavities were very narrow, causing the sound waves to mostly go through normal brain tissue as seen in Fig. 6 of patient 3.

### Quantitative image analyses

**Fig. 7:**
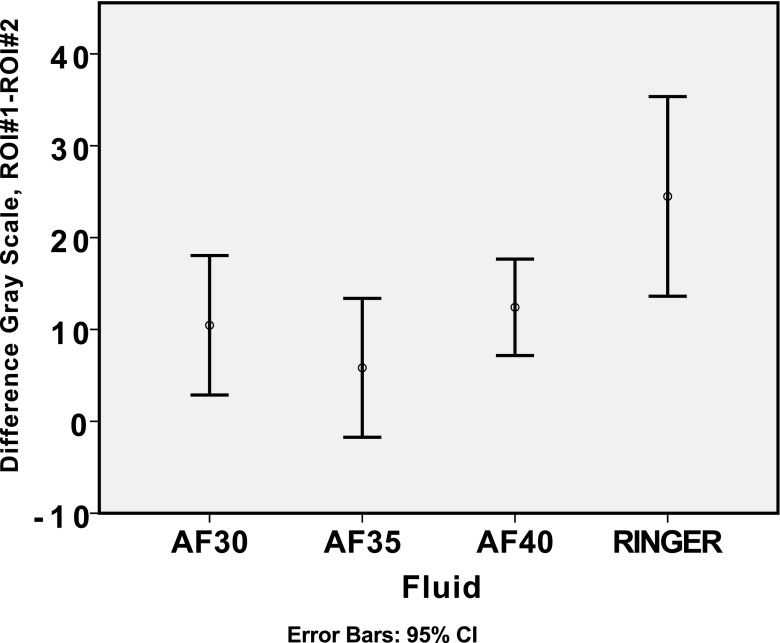
Differences in median grey level in region below resection cavity (ROI #1) and reference tissue region (ROI #2). The average difference is indicated with a circle, and error bars indicate 95% confidence interval

The median voxel intensity in a region below the resection cavity (ROI #1) and a reference region (ROI #2) laterally to the resection cavity was calculated for each of the ultrasound image volumes. In Fig. 7, the difference in the median values between ROI #1 and ROI #2 is plotted. The fluid ACF-2 is having the lowest average difference in intensity between the selected regions of interests for the ACF fluids, while Ringer’s solution has the highest average difference. A lower difference indicates an intensity level below the resection cavity being more similar to the intensity level of the reference region, whereas a higher and positive difference in pixel brightness level will indicate an artificial signal enhancement in the region below the resection cavity.

All series of differences ROI #1–ROI #2 for each fluid were normally distributed (Kolmogorov-Smirnov, *p* > 0.2), and consequently *t* test (one-sample) was used for the statistical analyses. The statistical analyses show that the images obtained with Ringer’s solution, ACF-1 and ACF-3 have differences significantly greater than zero (*p* < 0.01). For images obtained with ACF-2, the null hypothesis of zero difference cannot be rejected (*p* > 0.1).

### Postoperative adverse events and clinical follow-up

The postoperative adverse events were found by inspection of patient journals, discharge reports, MR imaging and interviews with the patients. A few patients refused or were too exhausted to participate in some of the scheduled controls, interviews and/or MRI investigations during the 6-month study period. Accordingly, follow-up data are missing in these patients.

Early postoperative (24–72 h) MR images did not indicate any inflammation or adverse effect in the brain tissue, nor any significant circulatory changes or dilated ventricles in any of the 15 patients included in the study. Neither did the MR examination performed in 13 patients 1 month after the operation indicate any inflammation of tissue or dilated ventricles. In 12 patients having an MR examination at 6 months postoperatively, one of the patients was measured to have dilated ventricles. None of the patients had any indication of inflammation of tissue. Tumour recurrence was found in 5 of 12 patients (42%) at the 6-month follow-up.

In Table 3, there is an overview of the adverse events found by the postoperative controls and by examination of digital patient journals. One patient had fever (38.6 °C) and neck stiffness occurring 6 days after the surgery, along with a CSF leakage causing subcutaneous accumulation. Samples from the subcutaneous CSF were acquired along with samples obtained by lumbar puncture. The CSF samples did not imply any bacterial growth. The CSF of the lumbar samples contained erythrocytes. The CRP was normal.

**Table 3:** Postoperative events of the included patients

Type of postoperative event	Within 30 days No. (%)	Between 1 and 6 months No. (%)
Fever (unknown origin)	1 (7)*	1 (7)
CSF-leak	1 (7)	0 (0)
Epileptic seizures	2 (13)*	1 (7)
Epidural hematoma requiring surgery	1 (7)*	0 (0)
Epidural hematoma without clinical symptoms	1 (7)	0 (0)
UTI/urinary retention	3 (20)	0 (0)
Salmonella infection	1 (7)	0 (0)
Aloofness (episode of 15 min)	1 (7)	-
Subdural effusion	1 (7)*	0 (0)
Pneumonia	2 (13)	1 (7)
Itchy rash	2 (13)	0 (0)
Syncope (3 episodes)	1 (7)	0 (0)
Fungal mouth infection	1 (7)	0 (0)
Cardiac arrhythmia	0 (0)	1 (7)
Chronic subdural hematoma	0 (0)	1 (7)*
Febrile neutropenia	0 (0)	1 (7)
Death	0 (0)	3 (20)*

*Reported as serious adverse events (SAEs) or suspected unexpected serious adverse reaction (SUSARs) according to the ICH/GCP-guidelines

Two patients were reported to have seizures within 30 days after surgery. One patient had an epileptic seizure within 3 days after surgery. This patient had in the 3 weeks before the operation had three episodes with sudden aphasia lasting only a few seconds and three episodes with shivering of right hand lasting about 1 min. The patient was operated in the cingulate gyrus and some tumour tissue was intentionally left behind. The other patient, that was operated in the right sensory cortex and close to right pyramidal tract, had attacks with painful rigidity in the left leg starting 6 days after surgery. It was interpreted as a focal seizure. Antiepileptic drugs had very limited effect. None of the patients were given pre-and postoperative prophylactic antiepileptic drugs. There were two incidences of epidural hematoma, of which one required surgical evacuation.

**Fig. 8:**
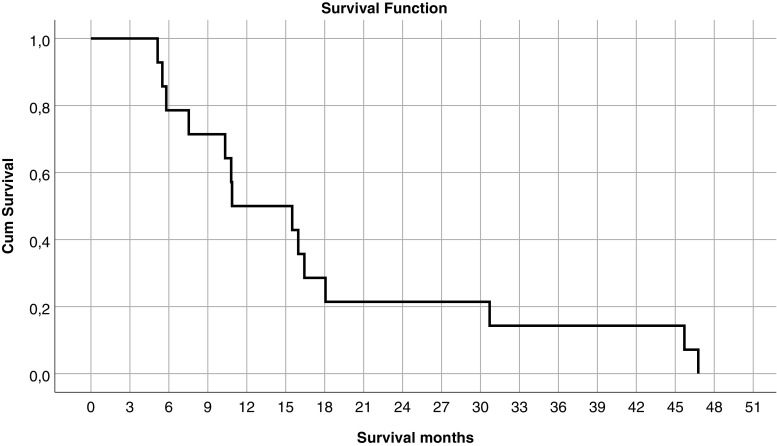
Cumulative survival curve for the enrolled glioblastoma patients

The median survival for the glioblastoma patients enrolled in the study was 326 days (10.9 months). Figure 8 shows the survival curve. Three of the 15 glioblastoma patients died between the 1-month and the 6-month follow-up period.

### Health-related quality of life (QoL), cognitive function and functional status

The patients reported their health-related QoL in the EQ-5D questionnaire. A minimal clinically important difference (MCID) of ± 0.15 was used for defining whether the patient’s QoL improved or deteriorated [9]. Compared to baseline 5 of 14 patients (36%) had deteriorated at 1 month and 5 of 11 patients (45%) at 6 months after surgery.

Cognitive function of the patients was evaluated using the mini mental status (MMS) and the cognitive functioning scale of EORTC QLQ-C30. For the functioning scale of EORTC QLQ-C30, a MCID of 10 points was used to evaluate improvement or deterioration relative to the status prior to surgery [5]. At 1 month after surgery, 11 patients answered the EORTC QLQ-C30 questionnaire. Four of them reported improved cognitive function (36%), four reported unchanged cognitive function (36%) and three reported deterioration (27%).

For the MMS, a drop of ≥ 2 compared to the preoperative score was registered in 9 of 14 patients immediately after surgery (24–72 h). At 1 month, three patients had a drop ≥ 2 compared to the preoperative score.

There was no drop in Glasgow Coma Scale neither immediately after surgery nor after 1 and 6 months. In NIH Stroke Scale 3 of 14 patients had a change > 2 immediately after the operation. At 1 month and 6 months after the operation, this change was registered in one patient.

The functional impairment was evaluated by a drop in KPS from ≥ 70 before surgery to ≤ 60 after surgery. Immediately after surgery (24–72 h), such a drop was registered in seven patients. One month after surgery, the number with such a drop compared to preoperative value was reduced to three patients.

## Discussion

The study has investigated the safety and image quality of a novel acoustic coupling fluid (ACF) intended to be used during ultrasound imaging in brain tumour surgery. Three concentrations of ACF were used in addition to Ringer’s solution, which served as the current gold standard.

The image quality for each of the ultrasound images obtained was assessed and rated by the surgeon in the operating room during the ultrasound imaging and in a blinded fashion postoperatively by five neurosurgeons with experience in ultrasound imaging. In addition, the impacts on noise of the three different ACF concentrations and the Ringer’s solution were investigated by quantitative analyses of the pixel brightness in defined ROI’s. All the analyses demonstrate that there is less noise in the group of ultrasound images obtained with ACF in the resection cavity compared to the images obtained with Ringer’s solution. The postoperative assessment of the ultrasound images indicates that the improved image quality will make it easier to identify remaining tumour in the area below the resection cavity. The improved ultrasound image quality with ACF may lead to better clinical decision support for the surgeons.

When considering the improvement in image quality for the individual patients, there was none of the cases where the use of ACF led to poorer image quality compared to Ringer’s solution. However, in two cases with small resection cavities, the postoperative analyses indicated a similar image quality between ACF and Ringer. This is in accordance with the theoretical background for the artefacts. As long as the sound waves go through tissue, there will not be enhancement artefacts. Therefore, the subjective interpretation of the degree of improvement in image quality will also depend on the size of the resection cavity.

When comparing the noise level observed in the three different concentrations of ACF, the differences were small compared to the differences between ACF and Ringer’s solution. The attenuation of the fluid is a balance between being too low (compared to attenuation in brain tissue) and thereby not reducing the noise as much as it could have done and being too high which could lead to shadowing artefacts in the ultrasound image below the resection cavity. For that reason, fluid with three different concentrations of the sound attenuating component was tested. The quantitative analyses of image contrast show that the ACF-2 images are most similar to the intensity of a reference region with similar tissue. The surgeon’s perioperative assessment of the different images acquired with ACF in the cavity also favours the same concentration (ACF-2).

There were three deaths within the 6-month study period. Two of the patients died approx. 5.5 months after surgery, with recurrent tumour apparent on the MRI obtained 1 month after surgery. The deaths were caused by tumour progression. One patient died 5 months after surgery. In this patient, it was not identified tumour recurrence on the 1-month MRI examination. The adjuvant treatment with radiation therapy and chemotherapy was stopped early after it was initiated, due to neutropenia and deteriorated health. Even if MRI has not verified tumour growth for this patient, the death of the patient was considered to be caused by tumour growth and factors related to the progression of the disease. Both overall survival and 6-month death rate for patients in this study are comparable to the results reported for a similar group of patients consecutively operated at the same hospital from 2011 to 2013 [10].

Postoperative MRI examinations have not revealed any signs of inflammations nor toxicity in the brain of the patients included in the study. MRI is known to be sensitive for detection of inflammation in the brain parenchyma [8]. This indicates that the ACF fluid is well tolerated by the brain parenchyma. Nor has it been found any circulatory changes in the early postoperative MR images.

Patient number 1 was suspected to have meningitis 6 days after surgery, with headache, fever and some neck stiffness. The CRP was normal and tests of the cerebrospinal fluid (CSF) did not show any bacterial growth. This patient was operated for a very large tumour that invaded the ventricles. The tumour was radically removed resulting in a large opening from the resection cavity into the ventricles, and some blood had inevitably entered the ventricle system. There was blood in the samples of CSF taken days after surgery. The blood in the ventricle system might therefore explain the fever episode and neck stiffness. It seems less likely that the use of ACF in the earlier stages of surgery before opening of the ventricles should have caused the above stated complication.

In the same patient, the radiologist reported enlarged ventricles as well as tumour growth at the 6-month control. The clinical follow-up did not reveal any clinical symptoms of hydrocephalus. The increased risk for hydrocephalus by entering the ventricle during tumour surgery is well known. For example, a paper from Henry Ford Hospital in Detroit reported 11% hydrocephalus in such patients [3].

Rates of postoperative seizures in patients with gliomas without preoperative seizures vary in the literature from 4 to 40% in the year following resection, with the first postoperative seizure generally occurring within the first month after craniotomy [7]. In a prospective randomised study of perioperative seizure prophylaxis from MD Anderson Cancer Center in Houston, the authors found new seizures during the 30-day postoperative period in 18.6% of 46 glioma operations (80% were high-grade gliomas) with no benefit of prophylaxis [20]. In a retrospective study of 184 glioblastoma patients, new onset epilepsy during the first 30 days after resection was found in 16% in the group without prophylactic antiepileptic medication [4]. The two patients registered to have postoperative seizures in our study are within the level of what has been reported by others. However, due to the low number of patients in our study and the large variation in postoperative seizures in the literature, we cannot exclude the possibility that ACF can contribute to the generation of new seizures.

One patient reported itchy rash on the first postoperative examination at 1 month. The condition was not treated, and the rash was not reported in the patient’s journal or the nursing log. Another patient had a rash on back/neck 2 days after surgery that was reported as a possible urticaria. Some medication was altered; the patient was given antihistamine (cetirizin) and the rash went away the same day.

### Quality of life, cognitive function and functional status

The patient reported quality of life with deterioration found in 36% and 45% at 1 month and 6 months respectively after surgery was similar to the values found in a study of comparable patients operated at the same hospital from 2011 to 2013, and assessed in the same way [9].

In the early postoperative examination (24–72 h) of MMS, there was a drop. Postoperative cognitive dysfunction is a well-known entity [18] that to our knowledge has not been studied in the immediate postoperative period after brain surgery. It is likely, however, that the operation itself, and also the certainty of having an incurable tumour, will influence the ability of the patients to answer questions immediately after the operation.

The Karnofsky performance index also dropped immediately after surgery. We did not find results to compare with in the literature, but it is likely that the scoring immediately after a brain tumour operation will be influenced by the operation itself. In a month, the functional activity was regained in most of the patients.

Patients operated for glioblastoma have a high rate of postoperative adverse events [1]. This makes it difficult to identify adverse events related to the intervention. Even though the adverse events in this study are within the limits of what have been reported in other publications, indisputable safety data requires a higher number of patients operated using ACF in ultrasound-guided surgery.

## Conclusion

In a phase I study of glioblastoma patients, a novel acoustic coupling fluid (ACF) was able to remove artefacts that appear in ultrasound images towards the end of tumour removal. Our findings indicate that the improved ultrasound image quality with ACF may lead to better clinical decision support for the neurosurgeons.

Adverse events or complications detected in this limited group of patients are comparable to what has been reported for similar patients in other publications.
